# Intrauterine﻿ growth restriction weakens anticontractile influence of NO in coronary arteries of adult rats

**DOI:** 10.1038/s41598-021-93491-3

**Published:** 2021-07-14

**Authors:** Ekaterina K. Selivanova, Anastasia A. Shvetsova, Lyubov D. Shilova, Olga S. Tarasova, Dina K. Gaynullina

**Affiliations:** 1 Department of Human and Animal Physiology, Lomonosov Moscow State University, Moscow, Russia; 2 Institute for Biomedical Problems, Russian Academy of Sciences, Moscow, Russia; 3 Russian National Research Medical University, Moscow, Russia

**Keywords:** Physiology, Circulation

## Abstract

Intrauterine growth restriction (IUGR) is one of the most common pathologies of pregnancy. The cardiovascular consequences of IUGR do not disappear in adulthood and can manifest themselves in pathological alterations of vasomotor control. The hypothesis was tested that IUGR weakens anticontractile influence of NO and augments procontractile influence of Rho-kinase in arteries of adult offspring. To model IUGR in the rat, dams were 50% food restricted starting from the gestational day 11 till delivery. Mesenteric and coronary arteries of male offspring were studied at the age of 3 months using wire myography, qPCR, and Western blotting. Contractile responses of mesenteric arteries to α_1_-adrenoceptor agonist methoxamine as well as influences of NO and Rho-kinase did not differ between control and IUGR rats. However, coronary arteries of IUGR rats demonstrated elevated contraction to thromboxane A2 receptor agonist U46619 due to weakened anticontractile influence of NO and enhanced role of Rho-kinase in the endothelium. This was accompanied by reduced abundance of SODI protein and elevated content of RhoA protein in coronary arteries of IUGR rats. IUGR considerably changes the regulation of coronary vascular tone in adulthood and, therefore, can serve as a risk factor for the development of cardiac disorders.

## Introduction

Cardiovascular disorders are one of the leading causes of death and disability in developed countries. Increasing number of evidences is accumulating in favor of fetal origin of many cardiovascular diseases in adulthood^1^. Poor intrauterine supplying can change functioning of fetal cardiovascular system and, therefore, increase susceptibility to cardiovascular diseases in adulthood^2–4^.

One of the most common pathologies of pregnancy is intrauterine growth restriction (IUGR) that accompanies 5–15% of all pregnancies in the USA and Europe and even more in low-income countries^5^. There are a lot of different risk factors for the development of IUGR, including but not limited to maternal undernutrition, high altitude hypoxia, congenital infections, smoking and alcohol or drug abuse^6^. Accordingly, animal models have been developed to study IUGR caused by these different factors. The model of food restriction is widely used in rats to simulate maternal undernutrition^6^.

The increasing number of studies has revealed that cardiovascular consequences of IUGR do not disappear in adulthood and may manifest themselves as an increase in systemic blood pressure^7^ or dysregulation of vascular functioning^8^. Among the various mechanisms of vascular functioning affected by this pathology, several studies have shown the weakened reactions of agonist-induced endothelium-dependent relaxation due to reduced nitric oxide (NO) bioavailability^9–12^. However, the influence of IUGR on two vasomotor mechanisms that are usually affected in the development of cardiovascular diseases has never been addressed. These mechanisms include anticontractile influence of NO (weakening of arterial contraction due to tonic NO production by vascular endothelium)^13^ and procontractile influence of Rho-kinase^14,15^. Importantly, intrauterine pathologies (for example, maternal hypothyroidism) can affect these pathways in offspring in early postnatal period^16^ or in adulthood^17,18^.

Thus, this study tested the hypothesis that that IUGR weakens anticontractile influence of NO and augments procontractile influence of Rho-kinase in arteries of adult offspring. To test this hypothesis, we utilized food restriction model of IUGR and studied the contribution of NO and Rho-kinase to the regulation of contractile responses of mesenteric and coronary arteries. The choice of mesenteric arteries is due to the fact that they are often investigated as representative arteries of systemic circulation including studies on adult IUGR animals. Coronary arteries, to the best of our knowledge, have not been studied in adult IUGR animals before, though they supply blood to such a vital organ as the heart. Of note, in fetal and neonatal coronary arteries IUGR enhanced contractile responses to agonists and reduced NO bioavailability^19,20^, pointing to the possible lifelong implications for the cardiac circulation.

## Results

### Characteristics of IUGR model

**Figure 1:**
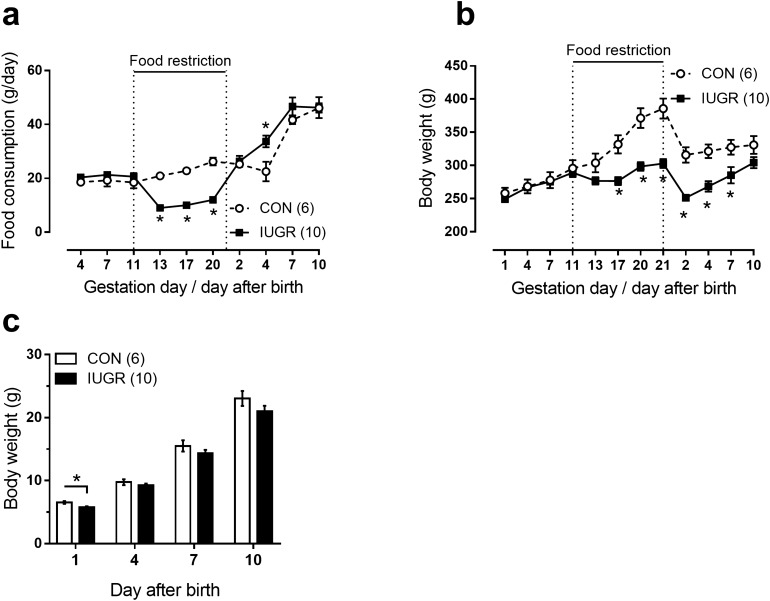
Food restriction during pregnancy (starting from the 11th gestational day till delivery) causes the decrease of dam body weight and the development of IUGR in their offspring. (**a**,**b**) Dam food consumption (**a**) and body weight (**b**) during gestation and first days after delivery in control and IUGR groups. (**c**) Pup body weight during first postnatal days in control and IUGR groups. Numbers in parenthesis indicate the number of females (**a**,**b**) or litters (**c**). *p < 0.05 (unpaired Student’s *t* test).

Food consumption by IUGR rats during the period of food restriction was significantly lower compared to control rats, however, after the termination of the restrictions the food consumption was elevated in IUGR group compared to control dams (Fig. 1a). Body weight of pregnant dams did not differ between control and IUGR groups from the gestational day (GD) 1 till the GD13. Starting from GD17 the body weight of dams from IUGR group was significantly lower compared to control dams (Fig. 1b). Body weight of food-restricted dams remained lowered during 1st postpartum week (Fig. 1b), despite the termination of food restriction immediately after delivery. The duration of gestation was 22 days for control dams and 23 days for food-restricted dams. The number of pups per litter before culling did not differ between groups (10.5 ± 0.6 for control group and 11.0 ± 0.6 for IUGR group, p > 0.05). On the first day after delivery, the number of pups in each litter was limited to 8.

Body weight of pups from IUGR group was decreased at postnatal day 1 compared to control pups, but did not differ between groups at postnatal days 4, 7, and 10 (Fig. 1c). At postnatal day 11 IUGR pups were characterized by smaller length of body (83.9 ± 0.7 mm (n = 16) vs. 85.9 ± 0.4 mm (n = 16) for IUGR and control groups, respectively, p < 0.05, measured from the tip of the nose to the base of the tail) and length of tail (34.8 ± 0.7 mm (n = 16) vs. 37.0 ± 0.7 mm (n = 16) for IUGR and control groups, respectively, p < 0.05).

**Figure 2:**
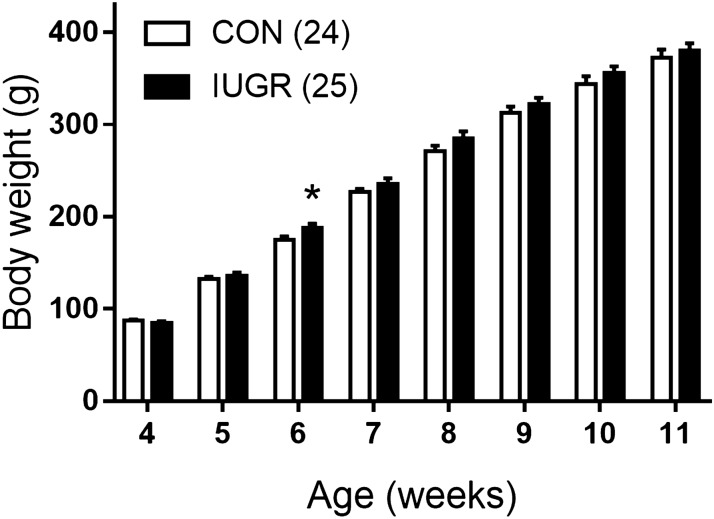
Body weight of male offspring from control and IUGR groups. Numbers in parenthesis indicate the number of animals. *p < 0.05 (unpaired Student’s *t* test).

After weaning at 4 postnatal weeks the male offspring body weight was similar in two groups of rats except postnatal week 6, where IUGR offspring demonstrated slightly increased body weight (Fig. 2). At the age of 12–13 weeks the blood levels of glucose, cholesterol as well as LDL and HDL did not differ between two animal groups (Table 1).

**Table 1:** Blood serum levels of energy substrates in adult rats from control and IUGR groups.

	Control	IUGR
Glucose, mM (n = 12; 13)	6.4 ± 0.3	5.9 ± 0.3
Cholesterol, mM (n = 12; 12)	1.86 ± 0.04	1.82 ± 0.08
Low density lipoproteins, mM (n = 12; 12)	0.16 ± 0.03	0.19 ± 0.04
High density lipoproteins, mM (n = 12; 12)	1.15 ± 0.05	1.12 ± 0.06

Numbers in parenthesis indicate the numbers of animals.

### The effects of IUGR on mesenteric arteries of the offspring

**Figure 3:**
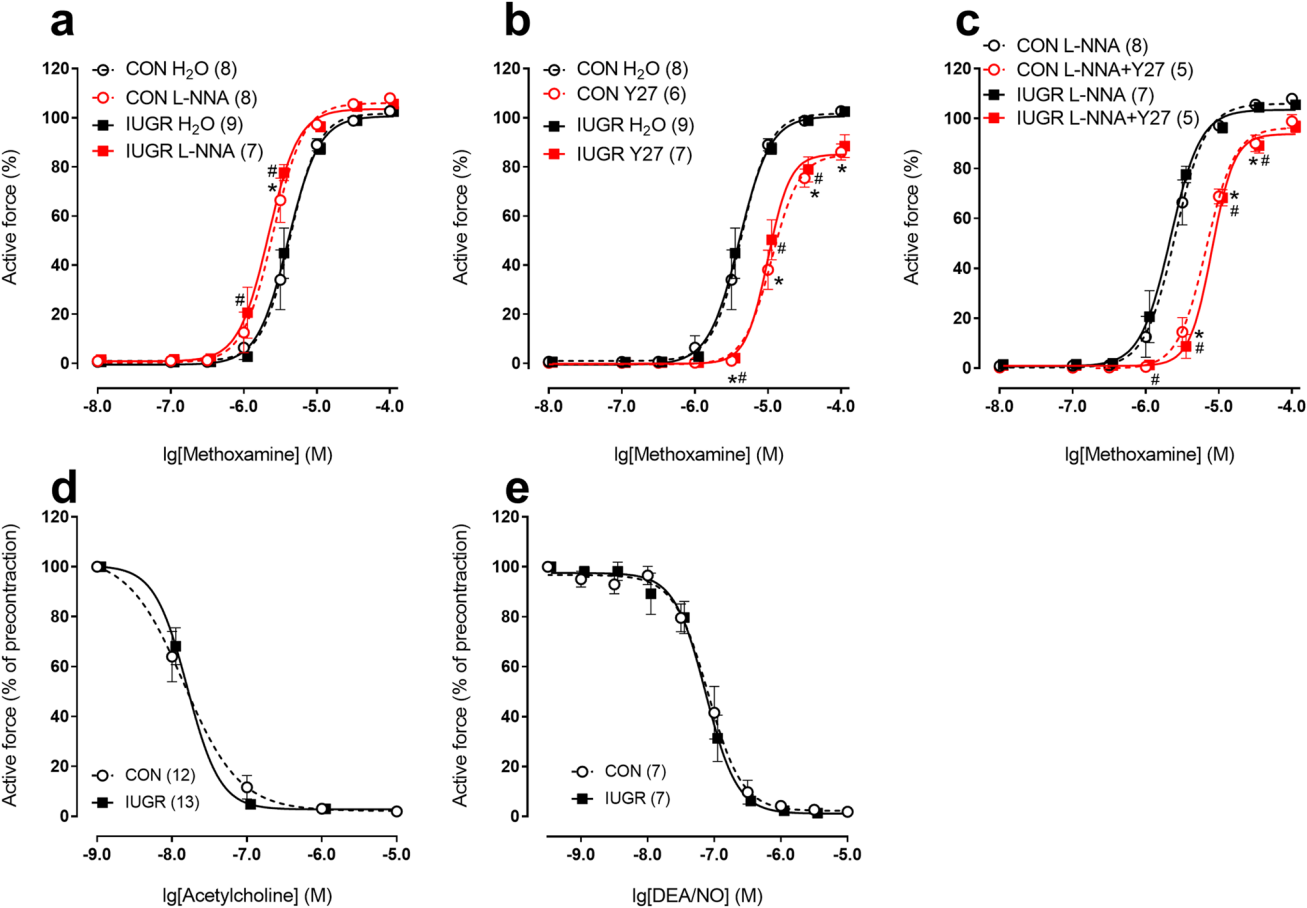
IUGR does not change the reactivity of mesenteric arteries in adult offspring. (**a**–**c**) Concentration–response relationships to methoxamine of mesenteric arteries from control and IUGR groups in the presence of vehicle (H_2_O) or 100 µM NO-synthase inhibitor L-NNA (**a**), vehicle (H_2_O) or 3 µM Rho-kinase inhibitor Y27632 (**b**), 100 µM L-NNA alone or in combination with 3 µM Y27632 (**c**). (**d**,**e**) Concentration–response relationships to acetylcholine (**d**) and NO-donor DEA/NO (**e**) of mesenteric arteries from control and IUGR groups. Data in (**e**) were obtained in the presence of 100 µM L-NNA. Numbers in parenthesis indicate the number of animals. *p < 0.05 for control group vs. H_2_O (**a**,**b**) or vs. L-NNA (**c**). ^#^p < 0.05 for IUGR group vs. H_2_O (**a**,**b**) or vs. L-NNA (**c**) (two-way ANOVA).

In mesenteric arteries, the relaxed inner diameter (d_100_, see “Methods” for description) as well as the isometric contractility (maximum active force) did not differ between IUGR and control rats (Table 2). The contractile responses to selective agonist of α_1_-adrenoceptors methoxamine (MX) were similar in control and IUGR groups (Fig. 3a). To estimate the anticontractile influence of NO, we studied the arterial contractile responses in the presence of NO-synthase inhibitor N^ω^-nitro-L-arginine (L-NNA). In both groups, L-NNA increased the responses to MX compared to vehicle-treated preparations (Fig. 3a). Accordingly, areas under curves (AUC) increased to 121 ± 6% in control group (n = 8) and to 125 ± 5% in IUGR group (n = 7, p > 0.05) (for non-normalized data see Table 2). In the presence of L-NNA the contractile responses to MX were similar between animal groups (Fig. 3a, Table 2).

**Table 2:** Relaxed inner diameter (d_100_), maximum active force and AUC for mesenteric and coronary arteries of control and IUGR rats.

Parameters	Control	IUGR
**Mesenteric arteries**		
Diameter d_100_, µm (n = 13; 13)	366 ± 13	374 ± 20
Maximum active force, mN (n = 13; 13)	18.3 ± 1.5	21.1 ± 0.9
AUC in the presence of H_2_O (a.u., n = 8; 9)	141 ± 10	143 ± 7
AUC in the presence of L-NNA (a.u., n = 8; 7)	170 ± 9	179 ± 8
AUC in the presence of Y27632 (a.u., n = 6; 7)	79 ± 6	88 ± 8
AUC in the presence of L-NNA + Y27632 (a.u., n = 5; 5)	112 ± 6	110 ± 4
**Coronary arteries**		
Diameter d_100_, µm (n = 13; 13)	279 ± 21	342 ± 9 *
Maximum active force, mN (n = 13; 13)	8.4 ± 0.9	10.9 ± 0.8 *
AUC in the presence of H_2_O (a.u., n = 8; 9)	126 ± 18	183 ± 16 *
AUC in the presence of L-NNA (a.u., n = 8; 8)	291 ± 25	318 ± 14
AUC in the presence of Y27632 (a.u., n = 7; 7)	48 ± 14	60 ± 8
AUC in the presence of L-NNA + Y27632 (a.u., n = 5; 5)	129 ± 23	114 ± 8

*p < 0.05 vs. respective value in control group (unpaired Student’s *t* test). Numbers in parenthesis indicate the numbers of animals.

Rho-kinase inhibitor Y27632 reduced contraction of mesenteric arteries in both control and IUGR rats (Fig. 3b), AUC decreased to 56 ± 4% in control group (n = 6) and to 62 ± 6% in IUGR group (n = 7, p > 0.05, Table 2). In the presence of Y27632 the responses were not different between control and IUGR groups (Fig. 3b; Table 2). In order to evaluate the contribution of Rho-kinase pathway under the conditions when NO-pathway is switched off, we applied Y27632 in the presence of NO-synthase inhibitor L-NNA. Under such conditions, arterial responses were similarly reduced in the two groups (Fig. 3c), AUC decreased to 66 ± 4% in control group (n = 5) and to 61 ± 2% in IUGR group (n = 5, p > 0.05, Table 2). Thus, Rho-kinase inhibitor Y27632 equally reduced contractile responses of mesenteric arteries in control and IUGR groups both in the absence and in the presence of NO-synthase inhibitor.

The endothelium-dependent relaxations of mesenteric arteries to acetylcholine were not different between control and IUGR rats (Fig. 3d), as well as the responses to NO donor diethylamine NONOate diethylammonium salt (DEA/NO, Fig. 3e).

### The effects of IUGR on coronary arteries of the offspring

**Figure 4:**
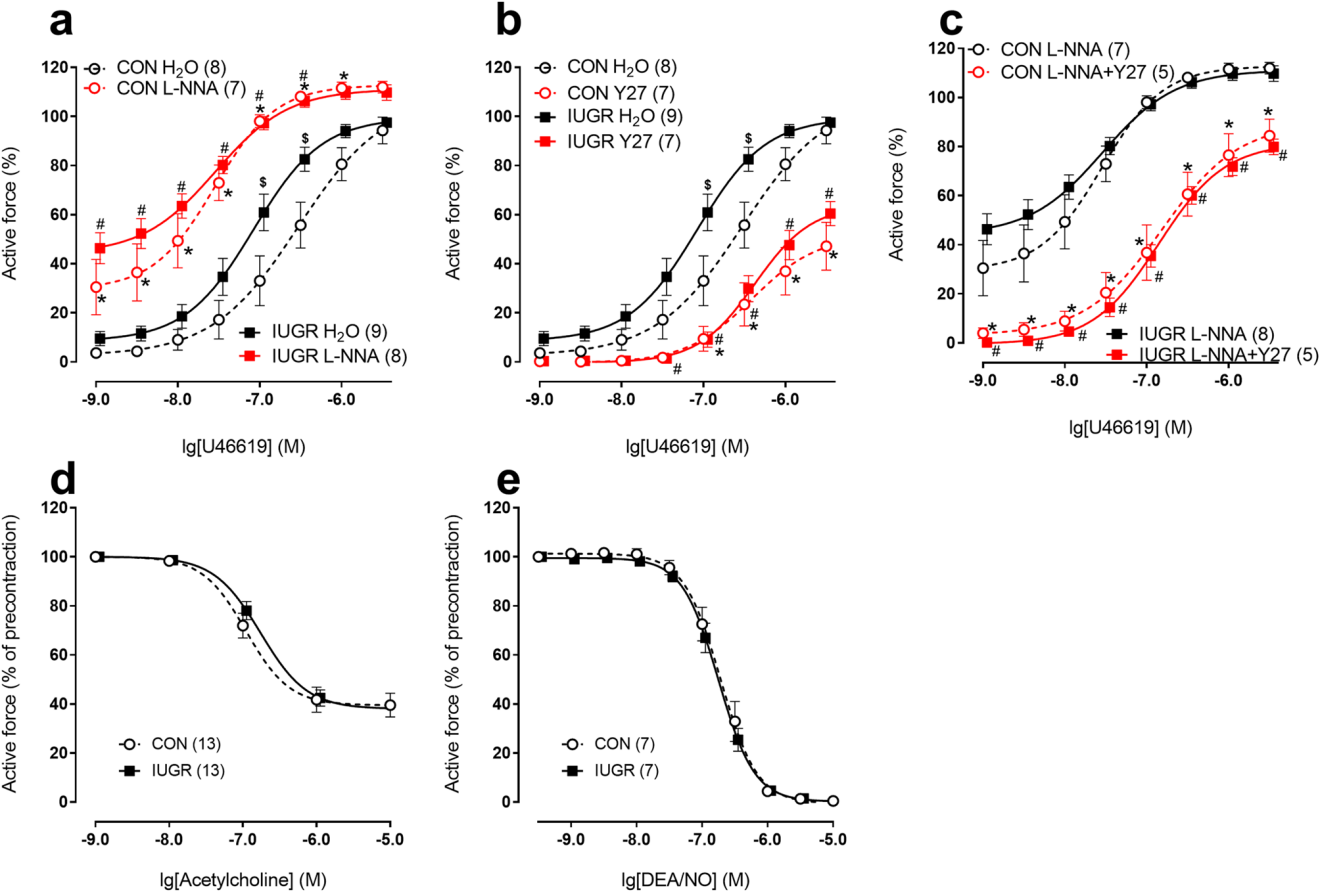
IUGR leads to the increase of contractile responses of adult offspring coronary arteries due to reduction of anticontractile influence of NO. (**a**–**c**) Concentration–response relationships to U46619 of coronary arteries from control and IUGR groups in the presence of vehicle (H_2_O) or 100 µM NO-synthase inhibitor L-NNA (**a**), vehicle (H_2_O) or 3 µM Rho-kinase inhibitor Y27632 (**b**), 100 µM L-NNA alone or in combination with 3 µM Y27632 (**c**). (**d**,**e**) Concentration–response relationships to acetylcholine (**d**) and NO-donor DEA/NO (**e**) of coronary arteries from control and IUGR groups. Data in (**e**) were obtained in the presence of 100 µM L-NNA. Numbers in parenthesis indicate the number of animals. *p < 0.05 for control group vs. H_2_O (**a**,**b**) or vs. L-NNA (**c**). ^#^p < 0.05 for IUGR group vs. H_2_O (**a**,**b**) or vs. L-NNA (**c**). ^$^p < 0.05 for control vs. IUGR group under respective conditions (two-way ANOVA).

In coronary arteries, the relaxed inner diameter (d_100_) and maximum active force were increased in IUGR rats compared to respective values in control (Table 2). Contractile responses to thromboxane A2 receptor agonist U46619 were augmented in IUGR rats compared to control rats (Fig. 4a; Table 2). L-NNA augmented the responses in both groups (Fig. 4a), AUC increased to 230 ± 20% in control group (n = 7) and to 174 ± 8% in IUGR group (n = 8, p < 0.05, Table 2). Importantly, in the presence of L-NNA the contractile responses were no longer different between animal groups (Fig. 4a; Table 2), that goes in accordance with significantly smaller increase of AUC in IUGR group compared to control one. These data indicate that in coronary arteries of IUGR rats the anticontractile influence of NO was diminished compared to control animals.

Y27632 reduced contractile responses of coronary arteries in both control and IUGR rats (Fig. 4b). Accordingly, AUC decreased to 38 ± 11% in control group (n = 7) and to 33 ± 4% in IUGR group (n = 7, p > 0.05, Table 2). Importantly, initially different contractile responses of coronary arteries did not differ after treatment with Y27632 (Fig. 4b). Along with that, similar contractile responses of L-NNA-treated coronary arteries were equally reduced by combined treatment with L-NNA + Y27632 (Fig. 4c, AUC decreased to 46 ± 8% in control group (n = 5) and to 36 ± 2% in IUGR group (n = 5, p > 0.05), Table 2).

Similar to mesenteric arteries, in coronary arteries the endothelium-dependent relaxations to acetylcholine and the responses to DEA/NO were not different between control and IUGR groups (Fig. 4d, e).

**Figure 5:**
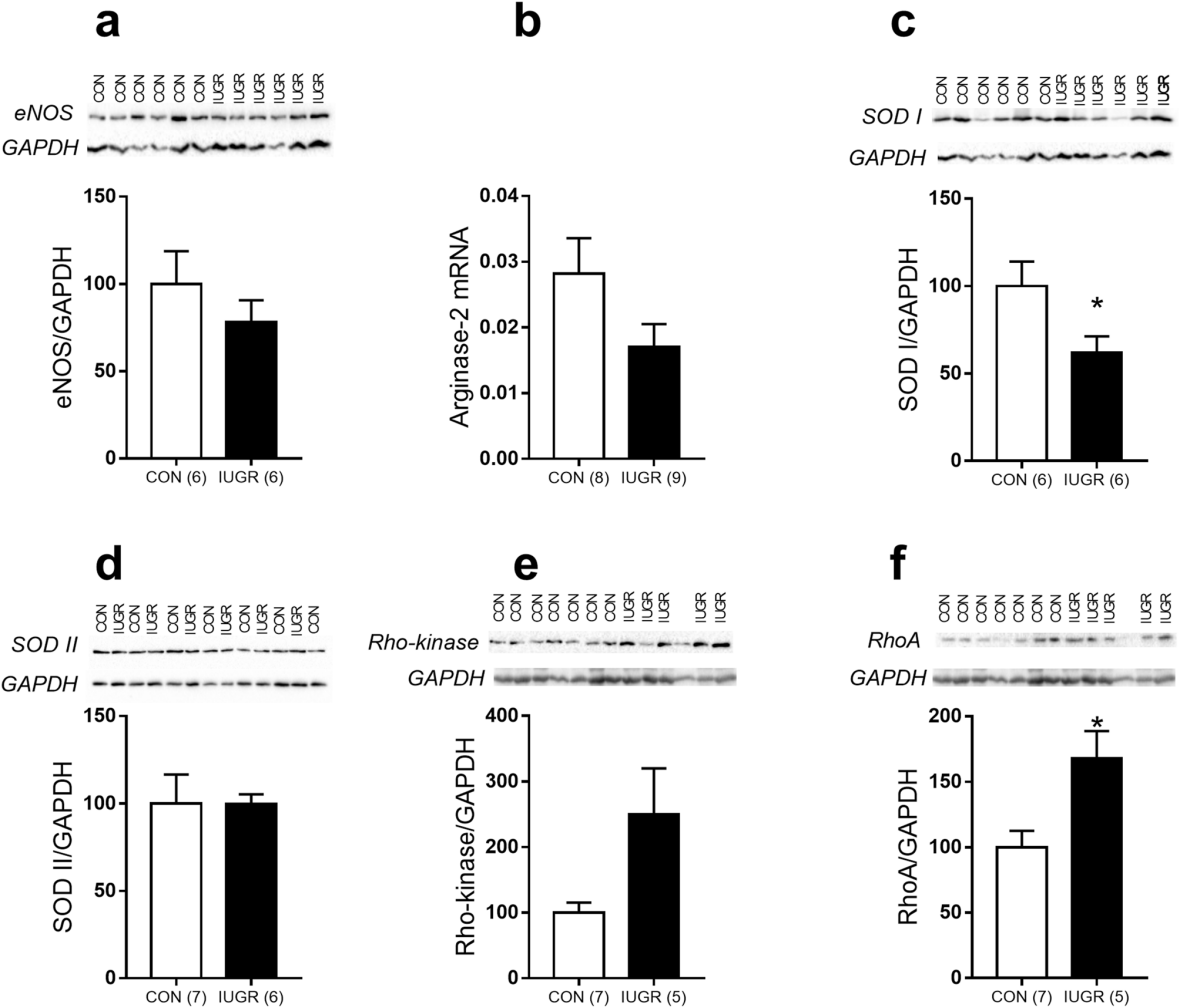
Protein and mRNA expression levels of regulatory molecules participating in NO- and Rho-kinase- pathways in coronary arteries of control and IUGR rats. (**a**,**c**–**f**) Protein abundance of eNOS (**a**), SOD I (**c**), SOD II (**d**), Rho-kinase (**e**) and RhoA (**f**). Data were normalized to GAPDH level in the same sample and average ratio in control group was taken as 100%. (**b**) Arginase-2 mRNA level in coronary arteries of control and IUGR animals. Data were normalized to the geometric mean of the two housekeeping RNAs (Gapdh and Rn18s), detected in the same sample and expressed as the percentage of the mean value of the control group. Numbers in parenthesis indicate the number of samples. *p < 0.05 (unpaired Student’s *t* test).

As the next step, we compared the abundance of molecules regulating the activity of NO- and Rho-kinase signaling pathways in coronary arteries of control and IUGR rats. The contents of eNOS and SODII proteins, as well as arginase-2 mRNA levels were not different between coronary arteries of control and IUGR rats (Fig. 5a, b, d). However, the content of SODI protein was reduced in coronary arteries of IUGR rats compared to control ones (Fig. 5c). The abundance of Rho-kinase protein was somewhat higher in arteries of IUGR rats (p = 0.09, Fig. 5e), while the abundance of RhoA protein was significantly elevated in arteries of IUGR rats compared to control group (Fig. 5f).

### Systemic cardiovascular effects of IUGR

In order to estimate the systemic production of NO we measured concentration of NO metabolites in blood serum of control and IUGR rats. The total content of nitrate + nitrite was not different between two studied groups (9.0 ± 0.8 µM in control group (n = 12) and 8.4 ± 0.7 µM in IUGR group (n = 12), p > 0.05). Further, the systolic blood pressure level was similar in two groups and comprised 138 ± 3 mm Hg in IUGR rats (n = 12) and 140 ± 2 mm Hg in control rats (n = 12) (p > 0.05), the heart rate was 423 ± 8 bpm in IUGR (n = 12) and 418 ± 13 bpm in control (n = 12) (p > 0.05).

## Discussion

Here we report novel findings that intrauterine growth restriction causes pronounced increase in coronary artery contraction due to reduction of NO-mediated anticontractile influence of the endothelium and augmentation of procontractile influence of Rho-kinase. These changes are specific for coronary circulation and apparently do not affect peripheral vascular beds, as seen from non-altered contractile responses of mesenteric arteries and systolic arterial pressure level in IUGR compared to control animals.

### Pups born to caloric restricted mothers demonstrate pronounced signs of IUGR

Caloric restriction during pregnancy is an established model of IUGR. In this study we utilized the model when 50% caloric restriction was applied to pregnant dams starting from GD11 till delivery, when required food amount is increasing considerably compared to the first half of gestational period. This model was successfully applied in several previous studies^21–23^. As expected, caloric restriction caused significant reduction of dam body weight. Pups born to caloric restricted dams demonstrated pronounced signs of IUGR, such as reduced linear sizes and body weight, similar to the literature data^21^. However, during post-weaning development the males of IUGR group no longer differed in weight from the control males, as previously described^24^. Furthermore, at the age of 3 months IUGR rats had normal level of blood serum parameters that characterize the metabolic profile of the organism. Normal metabolic state of rats born to caloric restricted mothers was previously observed in one-year old animals^25^. Thus, in our study adult progeny of food-restricted dams did not suffer from pronounced metabolic disorders that can serve as an additional risk factor for cardiovascular disease^26^.

### Regulation of mesenteric artery tone of adult animals is not altered by IUGR

Mesenteric vasculature are often used as a representative vascular bed of systemic circulation, including studies on IUGR animals^27–32^. In our study IUGR did not lead to the changes in maximum active force or diameter of mesenteric arteries. Furthermore, α_1_-adrenergic reactivity of these arteries was not changed by IUGR, that goes in accordance with previously published data^29,31,32^. Consequently, unaltered arterial responses in IUGR rats were not accompanied by apparent changes in the contribution of Rho-kinase or endothelial NO in the vasomotor control. Importantly, the NO signaling in vascular smooth muscle cells of mesenteric arteries explored by the responses to DEA/NO was not different between groups. This indicates that potential alterations in endothelial NO production in mesenteric arteries of IUGR group were not compensated by opposite alterations of NO-signaling in smooth muscle cells.

Endothelium-dependent dilations to acetylcholine were similar in mesenteric arteries of control and IUGR groups, which is supported by some previously reported data^29,30^. However, several other studies utilizing different animal models have revealed that IUGR can lead to endothelial dysfunction by reducing the contribution of NO to the reactions of agonist-induced endothelium-dependent arterial relaxation^9–12^. Of note, such endothelium-dependent relaxations and anticontractile influence of endothelium (when endothelium counteracts vasocontraction via tonically released NO, see^33^) represent two different aspects of endothelial functioning and can differentially change under pathologies affecting cardiovascular system^16,18,33–35^. Apparently, the same situation occurs in our IUGR model.

Taken together, our data demonstrate that the regulation of mesenteric artery tone in adulthood is not affected by maternal food restriction during the second half of gestation.

### Coronary artery contractile responses are strengthened in adult progeny of food-restricted dams

To the best of our knowledge, the control of coronary circulation has never been addressed before in adult IUGR rats, despite the key importance of this vascular bed. Our novel data show that the contractile responses of coronary arteries are augmented in adult IUGR rats due to reduced anticontractile influence of NO. Importantly, vascular smooth muscle reactivity to NO was not altered by IUGR, pointing to the endothelial origin of the decreased NO production/bioavailability. Notably, endothelium-dependent dilations to acetylcholine were not affected by IUGR, probably due to relatively weak contribution of NO to these reactions in rat coronary arteries^18^.

The next step of our study was to explore the mechanisms responsible for the attenuation of endothelial NO production or its bioavailability in coronary arteries of IUGR rats. We found that the abundances of eNOS and also Arginase-2, which competes with eNOS for L-arginine in vascular endothelium, were not altered by IUGR. However, the content of SODI, that reduces activity of reactive oxygen species thereby extending lifetime of NO, was decreased in coronary arteries of IUGR rats. Of note, decreased SOD1 expression along with endothelial dysfunction was observed in aorta of female rats with experimental preeclampsia^36^, in aorta and mesenteric arteries from high caloric diet-fed rats^37^, in skeletal muscle arterioles of aged rats^38^ and, importantly, in coronary arterioles from aged rats^39^. Thus, smaller abundance of SODI, but not SODII, can be one of the mechanisms responsible for reduced NO bioavailability in coronary arteries of adult IUGR rats.

We also addressed the potential role of Rho-kinase in modifying contractile responses of coronary arteries in IUGR rats. Of note, Rho-kinase was shown to participate in pathogenesis of vascular hypercontractility during hypertension, type II diabetes mellitus, coronary vasospasm, heart failure and other diseases^14,15^. However, the role of Rho-kinase in IUGR-induced disorders of coronary circulation has never been studied before, to the best of our knowledge.

Rho-kinase can augment vessel contraction by changing the functioning of both smooth muscle and endothelial cells^14^. In smooth muscle cells Rho-kinase inhibits myosin light chain phosphatase by either phosphorylation of its regulatory subunit or activation of CPI-17, a phosphatase inhibitor^40^. The key target of Rho-kinase in the endothelium is eNOS, Rho-kinase phosphorylates eNOS at Thr-495 thereby reducing its activity^41^. Therefore, to distinguish the role of Rho-kinase in smooth muscle and endothelial cells, we studied the effects of Rho-kinase inhibitor in the absence and in the presence of NOS inhibitor L-NNA. In the presence of L-NNA the effects of Rho-kinase inhibition did not differ in coronary arteries of adult control and IUGR rats, pointing to similar procontractile role of Rho-kinase in smooth muscle cells of two studied groups. However, in the absence of L-NNA the effects of the Rho-kinase inhibitor were more pronounced in coronary arteries of IUGR rats compared to control animals. Thus, procontractile role of Rho-kinase in the endothelium was higher in coronary arteries of IUGR rats, in line with higher abundance of Rho-kinase activating protein RhoA in their arteries.

Taken together, IUGR leads to augmented contraction of coronary arteries in adulthood by reducing the anticontractile role of NO and increasing procontractile role of Rho-kinase in endothelium. We suppose that reduced NO production/bioavailability in coronary arteries of IUGR rats is due to decreased SODI activity and also to increased Rho-kinase activity in endothelial cells.

### Regional differences in the effects of IUGR on arterial tone regulation

The results of our study show that IUGR can differently affect endothelium-dependent regulation of mesenteric and coronary vascular beds. Of note, different contractile agonists were used to induce contraction of mesenteric and coronary arteries, thus direct comparison between the two vascular regions must be done with caution. Nevertheless, our previous study proved that anticontractile influence of NO can be observed when either methoxamine or U46619 are used as contractile agonists^33^. In the present study we show that the anticontractile influence of NO is very different in mesenteric and coronary vascular regions. In control rats, the effect of L-NNA on contractile responses was much higher in coronary arteries compared to mesenteric ones, these observations go in accordance with previously published data^18,34^. Therefore, coronary circulation of adult rats is characterized by pronounced anticontractile influence of NO to large extent regulating the contraction of coronary arteries compared to mesenteric arteries.

Our results on systemic effects of our IUGR model suggest that most vascular regions of adult IUGR rats were not affected by IUGR, similar to mesenteric vasculature. The level of systolic blood pressure measured by tail-cuff plethysmography and NO systemic production estimated by serum concentration of nitrate + nitrite were not altered in IUGR rats. Of note, elevated blood pressure level in adult IUGR animals was shown previously in several studies^7,31,42,43^. Presumably, the discrepancy between the results of our and other studies could be due to the differences in IUGR models, severity of the disease or age of studied progeny^7,42,43^.

Thus, IUGR does not appear to change the vasomotor control of the most arterial beds of adult male rats. Along with that, IUGR considerably changes the regulation of their coronary circulation which does not make a decisive contribution to the total peripheral vascular resistance, but is critically important for the proper functioning of the heart. Importantly, similar studies should be carried out on females to assess whether the effects of IUGR on coronary circulation are sex-dependent, as was previously shown for blood pressure regulation^44^.

## Conclusion

Our novel data show that the anticontractile effect of NO is reduced in coronary arteries of adult IUGR rats. This is associated with augmented Rho-kinase pathway activity in endothelial cells of coronary arteries. Presumably, such alterations are specific for coronary circulation, since they were not observed in mesenteric arteries of rats born growth-restricted. Therefore, IUGR may disturb blood supply to the heart in adulthood and serve as a risk factor for the development of cardiac disorders.

## Methods

### Animals

Animal studies are reported in compliance with the ARRIVE guidelines. All experimental procedures used in this study were approved by Moscow State University committee on animal welfare (94-g) and conformed to the Guide for the Care and Use of Laboratory Animals published by the US National Institutes of Health (Eighth edition, 2011).

Adult female (230–280 g) and male (300–350 g) Wistar rats were obtained from the vivarium of the Institute of General Pathology and Pathophysiology (Moscow, Russia) and then bred in the laboratory animal unit of the Biological Faculty of Moscow State University. The animals were maintained on 12/12-h light/dark cycle and fed with normal rodent chow (containing 20% protein, 5% fat, calorie content 300 kcal/100 g) ad libitum.

### The model of intrauterine growth restriction

A model of IUGR was used where rat dams were 50% food restricted during the second half of gestation. Sexually mature males and females (age 2.5–3 months) were housed together for a night, at the next morning, the onset of pregnancy was determined by the presence of sperm in a vaginal smear (considered as the GD1). After that, the females were randomly assigned to the control (n = 6) and IUGR (n = 10) groups and placed in cages for individual maintenance. Food consumption, as well as body weight were regularly monitored. Dams of both the IUGR and control groups had unlimited access to water throughout gestation and postpartum periods.

From GD1 to GD10, females of both groups had unlimited access to chow. Starting from GD11, the females of the IUGR group received only half of food amount normally consumed by a pregnant female on the corresponding GD. From the day of delivery, the dams of the IUGR group obtained unlimited access to food. On the first day after delivery, the number of pups in each litter was limited to 8. After delivery, the weight of the dam and litter were regularly monitored. At the age of 11 days the linear body sizes of several pups randomly selected from each litter were measured.

Starting from the age of 4 weeks, the offspring of both groups of females were separated from their mothers and 2–4 males were randomly selected from each litter for further experiments. Offspring individual body weight was monitored weekly until the age of 11 weeks. At the age of 12–13 weeks the male rats were killed by decapitation under CO_2_ anesthesia and trunk blood was collected. Then small mesenteric arteries (2–3-order branches of the superior mesenteric artery) and septal coronary artery were isolated and used for wire myography experiments. Left coronary artery was isolated as well and used for Western blotting and qPCR experiments.

### Blood pressure measurements

At the age of 9–10 weeks the systolic blood pressure was determined in male offspring using tail-cuff plethysmography (Systola, Neurobotics, Russia) between 2 p.m. and 6 p.m. The rats were adapted to the experimental environment by performing the same procedures except blood pressure values collection. Four days later the systolic blood pressure level was recorded at least five times in each rat and the average value was taken into account. Heart rate values was obtained from plethysmography recordings as well.

### Blood samples analysis

Immediately after decapitation the blood glucose level was measured using Diacont express test strips. After blood clotting (20 min at room temperature followed by 40 min at 4 °C) the serum was separated by centrifugation for 15 min at 4300*g* and kept at − 20 °C till analysis. Total cholesterol serum concentration, high density lipoproteins (HDL) and low density lipoproteins (LDL) were determined using automatic biochemistry analyzer (A-25 Biosystems, Spain). The NO metabolites levels were measured using Griess method after reduction of nitrates to nitrites by VCl_3_^45^.

### Wire myography

Experiments on isolated arteries were performed as previously described^17^. Briefly, 2-mm-length segments of small mesenteric and septal coronary arteries were mounted on wires (with a diameter of 40 µm) and placed in myograph system (DMT, Denmark, models 410A or 620M) for isometric force recoding. The preparations were kept at 37 °C in physiological salt solution containing (in mM): 120 NaCl, 26 NaHCO_3_, 4.5 KCl, 1.2 NaH_2_PO_4_, 1.0 MgSO_4_, 1.6 CaCl_2_, 5.5 D-glucose, 0.025 EDTA, 5 HEPES, equilibrated with gas mixture 5% CO_2_ + 95% O_2_ to maintain pH = 7.4. Transducer readings were continuously recorded at 10 Hz sampling rate using E14-140 analog-to-digital data converter (L-Card, Russia) and PowerGraph 3.3 software (DISoft, Russia). The segments were gradually stretched to 0.9 d_100_, where d_100_ is the inner diameter of fully relaxed vessel exposed to the transmural pressure of 100 mmHg^46^. At the beginning of each experiment, mesenteric arteries were activated with noradrenaline (10 µM) and then with MX (10 µM), septal coronary arteries were twice activated with U46619 (1 µM). Endothelium-dependent relaxation was examined using acetylcholine (0.01–10 µM) applied on top of MX- or U46619-induced contraction (shown in Figs. 3d, 4d).

The main experimental protocol used in this study consisted of three cumulative concentration–response relationships (CRRs) to MX (0.01–100 µM, for mesenteric arteries) or U46619 (0.001–3 µM, for coronary arteries) separated by washout periods. First CRRs were performed in order to ensure similar initial responses of studied arterial segments. The second CRRs (shown in Figs. 3a,b, 4a,b) were obtained after 20-min incubation of one segment with NO-synthase inhibitor L-NNA (100 μM, Alexis Biochemicals) and the other one with equivalent volume of solvent (H_2_O, 50 µl). Twenty minutes before the third CRRs (shown in Figs. 3c, 4c), both segments were treated with Y27632 (Rho-kinase inhibitor, 3 µM, Calbiochem) in combination with either L-NNA or solvent, accordingly.

Responses to NO-donor were studied in the additional experimental protocol. After obtaining the CRR to MX or U46619 (for mesenteric and coronary arteries, respectively) the segments were incubated for 20 min with L-NNA (100 μM), in order to exclude the influences of endogenous NO. Then the segments were precontracted with MX or U46619 to 70–80% of the maximum active force and CRR to DEA/NO (0.001–100 μM, Sigma) was recorded (shown in Figs. 3e, 4e).

The wire myograph experiments were analyzed as described earlier^17^. All active force values were calculated by subtracting the passive force value (recorded in the preparation with fully relaxed smooth muscle) from all recorded values (before the first and at each agonist concentration). Then all active force values in MX- or U46619-induced CRRs were expressed as a percentage of the maximum active force value recorded in respective first CRR. The reactions to acetylcholine or DEA/NO were expressed as percentage of precontraction value. CRRs were fitted to a sigmoidal function with variable slope and AUC were calculated using GraphPad Prism 7.0 Software (La Jolla, CA). In order to compare the inhibitor effect between two groups of rats, AUC value in the presence of inhibitor was expressed as the percentage of average AUC value in the presence of solvent in the respective group.

### qPCR

qPCR experiments were performed similarly to previously described^47^. Briefly, left coronary arteries were isolated, immediately placed in RNA-later solution (Qiagen) and kept at − 20 °C pending further procedures. RNA was extracted using ExtractRNA kit (Evrogen, Russia) according to the manufacturer’s instructions. All RNA samples were processed with DNase I (Fermentas). RNA concentration was measured by a NanoDrop 1000 (Thermo Scientific, USA) and thereafter all samples were diluted to the same concentration. Reverse transcription was performed using the MMLV RT kit (Evrogen, Russia) according to the manufacturer’s protocol. qPCR was run in the RotorGene6000 using qPCRmix-HS SYBR (Evrogen). mRNA expression level was calculated as *E*^*−Ct*^, where *E*—primer efficiency and *Ct*—cycle number on which the curve for product accumulation is crossing the fluorescence detection threshold (calculated using the RotorGene6000 software). This value was normalized to the geometric mean of the two housekeeping genes (*Gapdh* and *Rn18s*), detected in the same sample. The expression level of *Gapdh* and *Rn18s* did not differ in coronary arteries of control and IUGR rats: E^−Ct^ for *Gapdh* was (6.13 ± 2.49) × 10^–8^ in CON group and (7.70 ± 2.25) × 10^–8^ in IUGR group (p > 0.05); E^−Ct^ for *Rn18s* was (2.37 ± 0.61) × 10^–4^ in CON group and (3.16 ± 0.85) × 10^–4^ in IUGR group (p > 0.05). Finally, the data were expressed as the percentage of the mean value of the control group.

Primers were synthetized by Evrogen and had the following sequences: *Gapdh* (forward: CACCAGCATCACCCCATTT; reverse: CCATCAAGGACCCCTTCATT), *Rn18s* (forward: CACGGGTGACGGGGAATCAG; reverse: CGGGTCGGGAGTGGGTAATTTG), *Arg2* (forward: CCAGCCTAGCAGTGGATGTGA; reverse: CTCTGGAATGCTGTCGTGAA).

### Western blotting

Western blotting experiments were performed similarly to previously described^17^. In brief, left coronary arteries were isolated and frozen in liquid nitrogen pending further procedures. To obtain one sample, 2 left coronary arteries from 2 animals were combined. Then they were homogenized in SDS-buffer (0.0625 mol/l Tris–HCl (pH 6.8), 2.5% SDS, 10% water-free glycerin, 2.47% dithiothreitol, 0.002% bromophenol blue) supplemented with protease and phosphatase inhibitors (aprotinin 50 mg/ml, leupeptin 100 mg/ml, pepstatin 30 mg/ml, NaF 2 mg/ml, Na_3_VO_4_ 180 mg/ml), centrifuged at 16,000*g* for 5 min at 4 °C; supernatant was kept at − 20 °C. Proteins were separated by SDS-PAGE and transferred to nitrocellulose membrane (Santa Cruz) using Trans-Blot Turbo transfer system (BioRad). The transfer was visualized with Ponceau S staining and the membrane was cut in three parts at the levels of appr. 28 and 75 kDa protein marker (Abcam) in order to reduce the quantity of antibodies used. All parts were blocked with 5% nonfat milk (Applichem, Germany) in TBS (20 mmol/l Tris–HCl, pH 7.6; 150 mmol/l NaCl) with 0.1% Tween 20 (TBSt). Then the lower part of the membrane was incubated overnight with antibodies against SOD I (Sigma-Aldrich, rabbit, 1:4000 in TBSt with 5% milk), SOD II (Enzo, rabbit, 1:1000 in TBSt with 5% milk) or RhoA (Abcam, rabbit, 1:2000 in TBSt with 5% milk). The middle part was incubated overnight with antibodies against GAPDH (Abcam, mouse, 1:2000 in TBSt with 5% milk). The upper part was incubated overnight with antibodies against Rho-kinase II (Abcam, rabbit, 1:5000 in TBSt with 5% milk) or eNOS (BD Transduction Lab, mouse, 1:2000 in TBSt with 5% milk). The next day, all membranes were incubated with appropriate secondary antibodies: anti-mouse (Cell Signaling, 1:5000 in 5% milk in TBSt) or anti-rabbit (Cell Signaling, 1:10,000 in 5% milk in TBSt) for 1 h and visualized with Super Signal West Dura Substrate (Thermo Scientific) using ChemiDoc (BioRad). Western blotting experiments were analyzed in ImageLab Software (BioRad). Protein of interest to GAPDH ratio was identified in each sample, and then the average ratio in control group was taken as 100%.

### Statistical data analysis

Statistical analysis was performed in GraphPad Prism 7.0. Unpaired Student’s *t* test or two-way ANOVA were used, as appropriate. Statistical significance was reached at p < 0.05. All data are given as mean ± SEM; n represents the number of animals or the number of samples in Western blotting experiments.

## Data Availability

The data that support the findings of this study are available from the corresponding author upon reasonable request.
